# The challenge of proving the existence of metazoan life in permanently anoxic deep-sea sediments

**DOI:** 10.1186/s12915-016-0263-4

**Published:** 2016-06-07

**Authors:** Roberto Danovaro, Cristina Gambi, Antonio Dell’Anno, Cinzia Corinaldesi, Antonio Pusceddu, Ricardo Cardoso Neves, Reinhardt Møbjerg Kristensen

**Affiliations:** Department of Life and Environmental Sciences, Polytechnic University of Marche, Via Brecce Bianche, 60131 Ancona, Italy; Stazione Zoologica Anton Dohrn, Villa Comunale, 80121 Naples, Italy; Department of Life and Environmental Sciences, University of Cagliari, Via Fiorelli, 1, Cagliari, Italy; Biozentrum, University of Basel, Klingelbergstrasse 50, CH – 4056 Basel, Switzerland; Natural History Museum of Denmark, Zoological Museum, Biosystematics Section, Universitetsparken 15, DK-2100 Copenhagen, Denmark

## Abstract

The demonstration of the existence of metazoan life in absence of free oxygen is one of the most fascinating and difficult challenges in biology. Danovaro et al. (2010) discovered three new species of the Phylum Loricifera, living in the anoxic sediments of the L’Atalante, a deep-hypersaline anoxic basin of the Mediterranean Sea. Multiple and independent analyses based on staining, incorporation of radiolabeled substrates, CellTracker Green incorporation experiments and ultra-structure analyses, allowed Danovaro et al. (2010) to conclude that these animals were able to spend their entire life cycle under anoxic conditions. Bernhard et al. (2015) investigated the same basin. Due to technical difficulties in sampling operations, they could not collect samples from the permanently anoxic sediment, and sampled only the redoxcline portion of the L’Atalante basin. They found ten individuals of Loricifera and provided alternative interpretations of the results of Danovaro et al. (2010). Here we analyze these interpretations, and present additional evidence indicating that the Loricifera encountered in the anoxic basin L’Atalante were actually alive at the time of sampling. We also discuss the reliability of different methodologies and approaches in providing evidence of metazoans living in anoxic conditions, paving the way for future investigations.

This paper is a response to Bernhard JM, Morrison CR, Pape E, Beaudoin DJ, Todaro MA, Pachiadaki MG, Kormas KAr, Edgcomb VG. 2015. Metazoans of redoxcline sediments in Mediterranean deep-sea hypersaline anoxic basins. BMC Biology 2015 13:105.

See research article at http://bmcbiol.biomedcentral.com/articles/10.1186/s12915-015-0213-6

## Background

The Deep-sea Hypersaline Anoxic Basins (DHABs) of the Mediterranean Sea are one of the most extreme oceanic realms known on Earth. The bottom sediments of these regions are completely anoxic and covered by a thick and dense brine (from tens to hundreds of meters), which hampers oxygen exchange. In particular, in the L’Atalante basin, the anoxic conditions are present since more than 50.000 years [1]. These conditions have been assumed for a long time to be excessively harsh to allow the life of multicellular eukaryotes, at least until the recent discovery of three new species belonging to Loricifera, a group of microscopic invertebrates (Metazoa). These three species were apparently able to live and complete their entire life cycle without access to free oxygen [2]. Using different and independent analyses based on incubations with radioactive tracers and specific fluorogenic probes (e.g. CellTracker Green), quantitative micro X-ray and infrared spectroscopy, and accurate analyses of different components of life cycles, Danovaro et al. [2] concluded that the loriciferans inhabiting the L’Atalante basin are metabolically active and show specific adaptations to the anoxic conditions. Furthermore, SEM and TEM analyses provided evidence that the cellular tissues were not degenerated.

Bernhard et al. [3], conducted an investigation in the same deep hypersaline anoxic basin (L’Atalante). Due to technical difficulties, the sampling of the bottom sediments, i.e., beneath the brines, was not possible. However, the authors collected sediment samples from the hypoxic redoxcline. Bernhard and coworkers found 10 specimens of Loricifera in the lower halocline, and only one from normoxic sediments (a specimen that resembles the recently described *Spinoloricus cinziae*; cf. [4]). They treated with DAPI (a fluorochrome used for DNA staining) the two Loricifera specimens (one from the halocline and one from the normoxic condition) and found that they were weakly stained. Moreover, the staining of the same individuals with Rose Bengal revealed the presence of a putative oocyte, but no other identifiable internal organs were observed. The presence of cadavers and animal remains (e.g., dead copepods and their exuviae) in the L’Atalante basin was pointed out by both teams [2, 3]. Bernhard et al. [3] suggested the possibility that benthic storms, namely those reported in the North-Western Mediterranean (which are ca 3000 km apart), could have transported the cadavers of the small crustaceans (and thus also the Loricifera) within the basin. However, invoking major physical processes to explain the presence of dead copepods in the system is not necessary since these very small organisms (size in the order of 150 μm) are able: (i) to swim in the boundary layer and thus they can be transported by deep-sea currents [5], and (ii) to enter in the system by simple sedimentation. In addition, there are no signs of storm events from the perfectly undisturbed and stratified sediments of the DHABs [6].

The analysis of SSU rRNA from the sediments of the halocline of the L’Atalante as well as of the normoxic sediments revealed the presence of a very low contribution of reads belonging to multicellular organisms, which were mainly represented by pelagic crustaceans. Nevertheless, Bernhard and collaborators could not find sequences of the nematodes that they reported as dominant taxon in all samples and, according to ultrastructural analysis, showed the presence of healthy tissue in normoxic sediments at the time of sampling. The authors also conducted *in situ* incubation experiments using CellTracker Green on normoxic sediments and on one sediment sample from the upper halocline of L’Atalante, but did not find Loricifera in their sample and thus could not obtain data from these experiments. However, they found labeled nematodes (indicating esterase activity at the time of incubation) and concluded that this reflected the nematode viability because no parasitic or scavenging prokaryotes were found on the nematode cuticle.

In our opinion, these results corroborate our previous findings [2] as we used the same analyses utilized by Bernhard et al. [3] to demonstrate the viability of nematodes in normoxic sediments as a proof of viability of the Loricifera in the permanently anoxic sediments of the L’Atalante basin (see below for a detailed analysis).

Moreover, the observation reported by Bernhard et al. [3] of living nematodes present at the oxic/anoxic interface (i.e., potentially moving actively in hypoxic-anoxic conditions) is interesting though not novel to science [7]. Organisms living at the normoxic/anoxic interface have been defined as *Thiobios* almost 50 years ago [8]. Interestingly, when the existence of these organisms was proposed, it was initially rejected: “*Thiobenthos does not exist*” [9]. Nowadays, the existence of the Thiobios (or *Thiobenthos*) is universally accepted by the scientific community [10], and in our opinion the loriciferans inhabiting the L’Atalante basin represent an example of the possibility of metazoan life in anoxic conditions.

The conclusions made by Bernhard et al. [3] diverge from those proposed by Danovaro et al. [2] in four main points. Bellow, we discuss the different methodologies and approaches utilized for providing evidence of the presence of metazoans living in anoxic conditions and compare our conclusions with those presented by Bernhard et al. [3].

### Evidence based on cell tissue staining

Danovaro et al. [2] pointed out that loriciferans found in the sediments of L’Atalante Basin could be perfectly stained with Rose Bengal, while all specimens of nematodes and copepods retrieved from the same sediment samples did not show or showed only a very weak staining. Furthermore, signs of the Rose Bengal staining in loriciferans was found in all their tissues/organs (e.g., brain, muscles, oocytes and epidermis cells).

In contrast, Bernhard et al. [3] suggested that the loriciferans stained with Rose Bengal were actually dead and the positive staining color was resulting from the presence of anaerobic bacteria, archaea and/or fungi living within the exoskeleton of the decaying loriciferans. However, this hypothesis should be ruled out because the investigation of some of the loriciferans found in the L’Atalante basin, and recently described as *Spinoloricus cinziae* [4], showed that specimens had very clean and non-decaying bodies. Indeed, a SEM analysis rendered images showing the absence of a single bacterium on the lorica or on the many spinoscalids of the head of the specimens investigated. This would not be possible if the animal has been dead. Furthermore, if the Rose Bengal staining was due to prokaryotes/fungi colonizing the degraded animal tissues, as suggested by Bernhard and co-workers, then a positive staining should be observed also in the dead nematodes and copepods found in the same samples.

All of the microscopic methodologies previously utilized (confocal laser microscopy, contrasting-phase microscopy, SEM and TEM) [2, 4] did not show any sign of degraded tissues, and the loriciferans were found either fully retracted, or partially retracted or fully extended (figures 7–11 in [4]). Moreover, since dead loriciferans are usually seen as fully extended and not stained by Rose Bengal, these features provide a good indication that these organisms were active at the time of sampling and responded to changes in the surrounding environment. Thus, although the Rose Bengal *per se* is not sufficient to prove that loriciferans were alive (as also reported by Danovaro et al. [2]), the criticism of Bernhard et al. [3] is not supported by the data presented. Therefore, according to available results, the loriciferans were collected alive.

### Evidence based on incorporation of radiolabeled substrates

Another technique utilized to provide evidence of active metabolism of loriciferans extracted from the anoxic sediments is the incorporation of radiolabeled organic substrates [2]. In contrast, Bernhard et al. [3] suggested that the radioactivity incorporated by loriciferans could be due to bacteria or archaea present within their body. In this regard, it is known that heterotrophic prokaryotes (either bacteria and archaea) can uptake leucine [11, 12]. However, the TEM investigations at the ultrastructural level of loriciferans from the L’Atalante basin by Danovaro et al. [2] provided evidence of the complete lack of abundant or aggregated prokaryotes, within the body of loriciferans. As reported above, SEM analyses also demonstrated the absence of prokaryotes in the lorica or in the many spinoscalids of the head providing evidence that the incorporation of radiolabeled leucine occurred within the tissues of loriciferans. Moreover, the magnitude of the radiolabeled substrate uptake makes highly improbable alternative explanations, even assuming a potential contribution from the symbiotic bacteria present within the animal tissues.

### Evidence based on metabolism (CellTracker Green labelling)

Bernhard et al. [3] stated that although esterase activity in loriciferans was clearly detected by CellTracker Green labeling performed by Danovaro and co-workers [2], bacteria also react to this fluorescent dye [13] and, hence, could produce the fluorescent reactivity observed inside the loriciferans. However, confocal laser microscopy analysis carried out on *Spinoloricus cinziae* (which was analyzed to the highest detail; [4]), revealed that the fluorescence was clearly present in different sections and parts of the body (from the head to abdomen and posterior lorica), and not only in specific parts of the body where the potential symbiotic bacteria were found. In addition, the conclusions made by Bernhard et al. [3] appear contradictory, because they incubated the nematodes collected from the halocline and normoxic samples with CellTracker Green and concluded that these nematodes were alive since they showed positive reactions. If Bernhard et al. [3] can state that this is a proof for the viability of nematodes, it should be as well a proof for the viability of the Loricifera incubated with CellTracker Green [2], which also showed the absence of parasitic organisms attached to their body.

### Evidence from molecular analyses

Extracting and sequencing RNA from living organisms can provide additional information on their ability to survive in anoxic conditions. Bernhard and co-workers [3] could not obtain any anoxic sediment sample below the brines of the DHAB (and thus any specimen of anoxic metazoans) to perform their molecular analyses.

Nor they were able to obtain rRNA sequences from any other taxon microscopically identified (mainly nematodes) both in normoxic and halocline sediments. All of the sequences of metazoans they found were affiliated to animal taxa belonging mainly to planktonic crustaceans.

The authors invoked four possible explanations: i) lack of primers’ specificity towards nematodes inhabiting the systems investigated; ii) an insufficient amount of sediment used for RNA extraction; iii) a nematode cuticle which does not protect the rRNA from degradation processes after nematode death and iv) the presence of signatures of pelagic copepods, which can have masked those of nematodes.

However, as far as the primer specificity is concerned, there is no reason to hypothesize that the primer pairs selected are not suitable for nematodes, since the region cover by that primers fall within the region we successfully amplified and sequenced from deep-sea nematodes [14]. Therefore, the primers utilized should have worked at least for the nematodes that Bernhard et al. [3] found in their normoxic sediments.

The nucleic acid extraction procedure carried out directly on a few grams of sediments that the authors used (i.e., *in situ* lysis approach) is known to be appropriate for molecular analysis of unicellular eukaryotes, but not for meiofauna, especially when very low abundances are encountered [15, 16]. This is confirmed by the fact that independently of the samples analyzed, including those from normoxic conditions, the largest majority of the sequences found were affiliated to benthic fungi and protists [17], with only a very small percentage of sequences affiliated to metazoans (0.02-6.5 %).

The conclusion that no nematodes were found due to decomposition processes of their RNA occurring post-mortem contradicts what the authors reported based on viability and ultrastructural analyses. This appears even more evident from the analysis of normoxic samples, where the authors identified living nematodes as well as in all other deep-sea sediments worldwide.

The isolation of nematodes from the sediments and the subsequent nucleic acid extraction are indispensable to avoid the masking effects related to the presence within the total RNA pools of sequences belonging exclusively to non-target organisms (as reported by Bernhard et al. [3]).

From the evidence provided by Bernhard et al. [3] we conclude that their analyses were biased in several aspects and thus could not allow any evaluation or proof of the presence/absence of living metazoans in their samples.

## Further insights into the characteristics of anoxic Loricifera

Besides the different interpretations discussed above, here we present further elements that in our opinion reinforce the evidence of anoxic metazoan life in the L’Atalante basin and could represent an important basis for future studies aiming at indentifying the presence of living metazoans in anoxic conditions.

### Presence of integer Loricifera in sediments dated 3000 years

One of the criticisms risen by Bernhard et al. [3] was based on the hypothesis that the Loricifera can be preserved (after their death) in anoxic conditions. However, we have two main evidence to rebut this hypothesis.

The first is that it is unreasonable that only loriciferans are preserved, while nematodes and copepods are not. The presence of nematodes and copepods in advanced decomposition, indeed, was also reported both by Bernhard et al. [3] in the lower halocline samples, and by Danovaro et al. [2] in the anoxic sediments of L’Atalante.

**Fig. 1.**
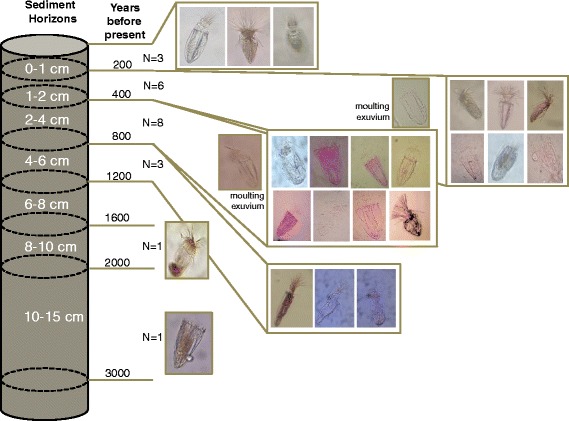
Vertical distribution of Loricifera in the anoxic sediments of the L’Atalante basin. The approximate age of sediment horizons (years before present) is provided as well as the images of some individuals found at different depths. The images show the presence of perfectly integer animals also in deeper sediment layers

The second reason to refute their hypothesis comes from the analysis we made of the vertical distribution of loriciferans in the anoxic sediments beneath the brine. We found loriciferans in different sediment horizons and also estimated the approximate age of the sediment horizons in which loriciferans were found [18]. The 10–15 cm sediment layer beneath the sediment surface, where loriciferans were found, can be dated approximately from 2000 to 3000 years before present (Fig. 1). Since we found exuviae and different integer loriciferans in different layers, it is unreasonable to conclude that some Loricifera can degrade in sediments 200–800 years old, while remaining preserved in sediments up to 3000 years older.

Moreover, the sediment layers of the anoxic basin are perfectly stratified [6], which allows hypothesizing the lack of any relevant physical disturbance caused by major sediment transport by turbidity currents [19]. Therefore, in our opinion the only likely explanation is the movement of living loriciferans into deeper sediment layers. An active movement of meiofauna (metazoans of size ranging from 20 to 500 μm to which Loricifera belong) across the sediments is an usual phenomenon in all sediments worldwide [10]. The body musculature of adult loriciferans account for muscle fibers related to the scalids and the mouth cone [20], which help to burrow deep in the sediments – even in the hadal zone, which is characterized by a high hydrostatic pressure [21].

### Additional evidence based on life cycle of Loricifera

Bernhard and co-workers casted doubts on whether the loriciferans are able to complete their life cycle in the anoxic sediments of the L’Atalante basin. They suggest that postlarvae exuviae could have reached the anoxic sediments like any other cadavers or moults. The single animal illustrated by Bernhard et al. [3] in figures 3a-3d, looks like a postlarva of *Spinoloricus* (the numbers of spinoscalid rows is 8 in postlarvae and 9 in adults; [22]), but it is very difficult to see the species characters from the available images. Moreover, the species belonging to *Spinoloricus* are very difficult to distinguish. In fact, *Spinoloricus neuhausi* was recently described from the Galapagos Spreading Zone after the reinvestigation of the type material of *Spinoloricus turbatio*, which is the type species for the genus and the first described from that region [22, 23]. Therefore, the *Spinoloricus* postlarva from an oxic zone could be substantially different from the postlarva of *Spinoloricus cinziae* that inhabits an anoxic zone. The first adults of *Spinoloricus* with well-developed ovaries were described from L’Atalante basin together with postlarvae and exuviae of postlarvae. These results reported for the first time the presence of nearly all stages involved in the life cycle of this organism in the same place, indicating thus that the species had molted in the DHAB basin [2, 4].

In addition, no other mature adult carrying an oocyte inside or post-larva has been reported from the entire Mediterranean basin before the study performed by Danovaro et al. in the DHAB L’Atalante [2]. The adult specimens found in the L’Atalante sediments were very healthy. Not only the oocytes in the ovaries could be observed, but also other internal structures, e.g. brain, midgut, and the external sensory structures known as scalids. Neves et al. [4] reported also different stages of body retractions. These details can only be observed if loriciferans are alive.

### Additional evidence based on the abundance of Loricifera

We present here further elements that contrast with the hypothesis of “cadavers” transported in the basin from surrounding normoxic sediments.

The average abundance of nematodes in the (oxygenated or normoxic) deep-sea sediments of the Mediterranean basin can be estimated as ca. 260,000 ind. m^−2^, whereas only 1,743 ind. m^−2^ were reported in the L’Atalante basin, where Danovaro and co-workers could only find cadavers, as indicated by their degraded tissues [2]. Therefore, the abundance of nematodes in the L’Atalante Basin is ca. 3 orders of magnitude lower than in normoxic sediments.

As far as Loricifera are concerned, Bernhard and co-workers reported a single specimen of loriciferans in normoxic sediments a few meters apart the halocline of the L’Atalante basin, and 10 individuals in the lower halocline [3]. From data reported by Danovaro et al. [2], it can be calculated that the abundance of loriciferans inhabiting this anoxic basin (ranging from 75 to 701 individuals per m^2^) is by far the highest abundance per unit of surface of the sediment investigated reported world-wide.

Moreover, on the basis of all meiofaunal samples analyzed so far over the last 30 years in the entire deep Mediterranean Sea (i.e., more than 500 samples) only in a few samples collected off Corsica, some undescribed individuals of loriciferans were observed (in the Western Mediterranean Sea, at ca. 3000 km distance from the L’Atalante basin; [24, 25]). Such a comparison indicates that the probability to find a single specimen of loriciferans in normoxic deep-sea sediments of the entire Mediterranean Sea is extremely low.

If the “cadavers fall” hypothesis (i.e., the downslope transport due to benthic storms or turbidity currents) would hold true, we should accept that the loriciferans are selectively transported into the anoxic basin with a rate of several orders of magnitude (5 to 6) higher than nematodes. These evidence allow us to reject this hypothesis.

### Additional evidence based on species richness and species distribution

One argument used by Bernhard and co-workers to cast doubts on the viability of loriciferans in anoxic sediments is that they found three morphotypes, one of them very similar to *Spinoloricus cinzae*, in the lower halocline of the L’Atalante basin [3]. However, the images provided are of insufficient quality to state whether or not the single loriciferan specimen found belongs to genus *Spinoloricus*. Furthermore, it would not be sufficient to demonstrate that these specimens could not live in anoxic sediments. First, because these organisms could have reached this zone by active mobility from the anoxic sediments beneath (a similar mechanism has been shown also for gobids in the hypoxic bottom of the upwelling region off Namibia; [26]. Second, Bernhard and co-workers reported that the three morphotypes of loriciferans were found in the sediments of the lower halocline (which is completely oxygen depleted below 2.5-cm depth) [3]. Therefore, there is no absolute evidence that the specimen encountered in the halocline was exposed to free oxygen.

Another important evidence in support of the presence of loriciferans inhabiting the anoxic sediments is related to their high biodiversity in anoxic conditions. The three species found in L’Atalante basin by both teams make the DHAB one of the most important biodiversity hotspot of Loricifera known worldwide. Only one coastal species, *Nanaloricus khaitatus*, has been previously described from the entire Mediterranean Sea [27]. Although undescribed deep-sea loriciferans are certainly present in the Mediterranean Sea [24, 28], the ecological and evolutionary model known as the ‘abundant centre’ hypothesis predicts that the higher the level of biodiversity, the higher the probability that species is originated from that system [29].

Along the line of this analysis and according to the evidence of data provided, it is incontestable that the loriciferans are much more common, diverse and characterized by the different life stages in the anoxic sediments than outside.

Our data lead us to hypothesize that the *Spinoloricus* found in the lower halocline by Bernhard and co-workers [3] could have originated from the anoxic sediment and spread up to the halocline (which is also anoxic below 2.5 cm depth) as a result of active movement.

## Conclusions

The conclusion of Bernhard et al. [3] that “*The possibility of viable metazoan community in the brines is not supported by our data at this time*” is correct, but is merely due to the fact that the authors could not: i) get samples from the permanently anoxic sediments beneath the brines, ii) perform metabolic analyses on Loricifera (e.g., CellTracker Green incorporation due to the lack of Loricifera in the samples incubated) and iii) obtain rRNA sequences of Loricifera. Demonstrating the presence of multicellular organisms with complex life cycles living permanently in an anoxic system is a grand challenge. Even a successful rRNA sequencing could be considered an insufficient proof of life in anoxic conditions, if we postulate that the organisms’ cadavers fallen into the basin from outside, and then remain preserved by the anoxic conditions. The only un-questionable evidence of life could be represented by a video (either *in situ* or in the laboratory) showing their mobility across different life stages and their feeding in anoxic conditions. However, this is not feasible at the moment with the available technologies. At the same time, the evidence provided by Danovaro et al. [2] remain un-confuted and the additional information reported here (e.g., the presence of integer Loricifera in sediments dated up to 3000 yrs before present, the presence of different stages of Loricifera life cycle only in anoxic sediments, their huge abundance and biodiversity in anoxic conditions), supports the hypothesis of existence of metazoan life in anoxic conditions. Future studies, are needed to understand the metabolic pathways and adaptation mechanisms, encoded in their genome, which allow these organisms to live in anoxic conditions.
